# Acquired and congenital disorders of sung performance: A review.

**DOI:** 10.2478/v10053-008-0068-2

**Published:** 2009-11-12

**Authors:** Magdalena Berkowska, Simone Dalla Bella

**Affiliations:** 1 Department of Cognitive Psychology, WSFIZ in Warsaw, Poland; 2 BRAMS Laboratories, Montreal, Canada

**Keywords:** singing proficiency, musical disorders, tone deafness, congenital amusia, song system, neurosciences of music

## Abstract

Many believe that the majority of people are unable to carry a tune. Yet, this widespread idea underestimates the singing abilities of the layman. Most occasional singers can sing in tune and in time, provided that they perform at a slow tempo. Here we characterize proficient singing in the general population and identify its neuronal underpinnings by reviewing behavioral and neuroimaging studies. In addition, poor singing resulting from a brain injury or neurogenetic disorder (i.e., tone deafness or congenital amusia) is examined. Different lines of evidence converge in indicating that poor singing is not a monolithic deficit. A variety of poor-singing "phenotypes" are described, with or without concurrent perceptual deficits. In addition, particular attention is paid to the dissociations between specific abilities in poor singers (e.g., production of absolute vs. relative pitch, pitch vs. time accuracy). Such diversity of impairments in poor singers can be traced to different faulty mechanisms within the vocal sensorimotor loop, such as pitch perception and sensorimotor integration.

## INTRODUCTION

Music, like language, is a typical human trait. Music-making (e.g., singing, playing an instrument, and dance) is a universal form of expression which is found in all societies and cultures (Mithen, 2006). For example, singing, rather than being the privilege of the few, is quite widespread in our society. People often sing when in group contexts (e.g., during religious ceremonies, in the military, at parties), but also when alone (e.g., humming the most recent pop hit). Participatory singing, typically held to be a very pleasurable experience, is likely to promote group cohesion (Mithen, 2006; Wallin, Merker, & Brown, 2000), thus fulfilling important social and communication functions (Welch, 2005). In contrast, poor singing, often treated as a hallmark of “tone deafness” or “unmusicality” (Sloboda, Wise, & Peretz, 2005), makes people less willing to participate in any forms of music-making (Clements, 2002).

The majority of individuals do not require formal vocal training or musical tutoring to sing proficiently. Like uttering first words and sentences, singing emerges spontaneously, and the ability to sing in tune and in time is mastered quite early during development. The impulse to sing is likely rooted in the universality of maternal singing (e.g., Trehub & Trainor, 1999), which is promptly imitated by infants. As a result, infants exhibit precocious singing abilities. During the first months of life, infants produce vocalizations (e.g., glissandi; see Papoušek, 1996), which can be seen as the precursors of music and speech intonation (Welch, 2005, for a review). The first meaningful vocalizations emerge by the end of the first year and include vowels sung at locally stable pitches. It is at around 18 months of age, however, that children produce recognizable songs (i.e., mostly short musical phrases repeated over and over; for reviews, see Dowling, 1999; Ostwald, 1973; and Welch, 2006). These first vocal productions contain the building blocks of adult singing, specifically stable pitch contour and regular beat patterns. Still, they lack stable tonality, which is achieved at around 5 years of age (Dowling, 1999; Dowling & Harwood, 1986). At that time, children already have a fairly large repertoire of songs from their own culture and, if they do not receive additional vocal training, their performances do not qualitatively differ from adult singing. Early singing skills pave the way for adult singing which is proficient in both pitch and time dimensions (Dalla Bella & Berkowska, 2009; Dalla Bella, Giguère, & Peretz, 2007), and remarkably consistent both within and between individuals (Bergeson & Trehub, 2002; Halpern, 1989; Levitin, 1994; Levitin & Cook, 1996). Thus, singing appears to be as natural as speaking for the majority.

With universality, early emergence, and orderly development, singing may fulfill some of the classic criteria for a complex human adaptation (e.g., Mithen, 2006; Wallin et al., 2000). Therefore, singing represents an invaluable source of information about the nature and origins of music. Yet, surprisingly, there is still a paucity of empirical studies on the psychological mechanisms underpinning singing in the majority (Gabrielsson, 1999; Parncutt & McPherson, 2002). Most research has targeted the acoustical properties of the singing voice in professional singers (e.g., Sundberg 1987, 1999). For example, particular attention has been devoted to the so-called *singer’s formant* (i.e., partials falling in the frequency range of 2.5–3.0 KHz; Sundberg, 1987), which in professional singers is much stronger in sung vowels than in spoken vowels. The intensity of the singer’s formant, the presence of vibrato, the maximum phonational frequency range, and loudness increase with musical experience (e.g., Brown, Rothman, & Sapienza, 2000; Hunter, Svec, & Titze, 2006; Mendes, Rothman, Sapienza, & Brown, 2003). Yet, just a few isolated studies have focused on the mechanisms underlying accurate pitch production in professional singers (Vurma & Ross, 2006; Zurbriggen, Fontenot, & Meyer, 2006). Zurbriggen and collaborators (2006) asked expert singers to prepare to sing a melody, which was produced in 50% of the cases but, in the remaining 50% of the cases, the singers were asked to switch to another melody. The accuracy of the first note of the melody and the melodic contour were affected in the different melody, thus suggesting that these elements are relevant in motor planning. In another study, Vurma and Ross (2006) showed that pitch intervals produced by professional singers can be out of tune by 20–25 cents, with respect to the equally tempered scale; this discrepancy went unnoticed by expert listeners judging performance accuracy. In summary, there is a bulk of evidence regarding vocal performance in professional singers, mostly regarding voice features. 

Unlike the vocal performance of experts, singing proficiency in laymen (i.e., whether everybody in the general population can sing in tune and in time) has been profoundly neglected. There are at least two reasons for this situation. First, there is a quite widespread belief that people without vocal training are generally inept at singing. This view is consistent with non-musicians’ self-assessment of their own singing proficiency (e.g., Pfordresher & Brown, 2007). Yet, it turns out that they are being too defeatist (Dalla Bella & Berkowska, 2009; Dalla Bella et al., 2007). The second reason pertains to methodology. Objective and quantitative assessment of pitch and rhythm accuracy in singing (e.g., via acoustical analysis) still poses some challenges and is typically very time-consuming. This situation contrasts, for example, with the analysis of piano performance, where keystroke onsets and offsets and key velocities can be accurately recorded via a computer-monitored MIDI-keyboard. It is not surprising, thus, to observe that most research in music performance has focused on piano playing, the output of which can be promptly recorded and analyzed using standard procedures.

The goal of the present article is to provide a review of the most recent experimental evidence on singing accuracy in the adult non-musician population. To this end, we will focus on singing accuracy in the pitch and time dimensions (i.e., whether the produced notes deviate in terms of pitch or duration from the target notes, as indicated by the notation). Although we are well aware that voice properties are relevant in judging whether somebody’s singing is “good” or “poor” (e.g., Himonides & Welch, 2006), this dimension will not be considered here. Hence, individuals termed *proficient singers* throughout this article may not necessarily be judged as such based on on their voice quality or on other features (e.g., microtonal variation). Results from behavioral and neuroimaging studies will be reviewed to characterize singing proficiency in the general population and elucidate its neuronal underpinnings. Attention will then be paid to cases of poor singing in non-musicians consequent to brain damage (i.e., acquired disorders) or resulting from life-long musical difficulties (i.e., tone deafness or congenital amusia, herein referred to as *congenital disorders*). Finally, building on this evidence, we will examine the mechanisms which are disrupted by a brain injury or brought to a halt during development, thereby leading to poor singing. The approach adopted is that typical of cognitive neuropsychology, where dissociations between symptoms in patients with brain damage or developmental disorders are taken as evidence reflecting the functional architecture of the normal brain (Rapp, 2001).

## NORMAL SINGING

There is a large amount of research on singing proficiency during development in music education (for a review, see Welch, 2006; see also Welch, 1979, for early studies on poor-pitch singing). Most research concerns children’s skills in imitating single pitches (i.e., pitch-matching tasks), intervals, or melodies. In these studies the effect on pitch accuracy of variables such as the model pitch, age, and perceptual skills was examined (see Demorest & Clements, 2007). For example, it was found that children can imitate female vocal models more accurately than male models (Green, 1990; Small & McCachern, 1983); moreover, pitch accuracy increases with age (Green, 1990; Klemish, 1974; Yarbrough, Green, Benson, & Bowers, 1991; Yarbrough, Karrick, & Morrison, 1995). Other studies compared perception and performance skills in accurate and inaccurate singing during development, yielding conflicting results. A strong link between pitch perception and production has been shown in some studies (Demorest, 2001; Demorest & Clements, 2007; Phillips & Aitchinson, 1997), but not confirmed by others (Apfelstadt, 1984; Geringer, 1983; Roberts & Davis, 1975).

The rich literature in the field of music education contrasts with the relatively scant evidence about singing proficiency in adults. Indeed, most believe that adults who have not received vocal training (i.e., occasional singers) are unable to carry a tune. This widespread view is confirmed by occasional singers’ judgments of their own sung renditions. For example, almost 60% of 1,000 university students reported that they cannot accurately imitate melodies (Pfordresher & Brown, 2007). Moreover, self-declared tone-deaf individuals (around 17% of the student population) believe that they cannot sing proficiently (Cuddy, Balkwill, Peretz, & Holden, 2005). Occasional singers, however, are likely to underestimate their actual singing skills. The prevalence of deficits affecting singing proficiency (e.g., poor-pitch singing) is lower, and probably confined to 10-15% of the population (Dalla Bella & Berkowska, 2009; Dalla Bella, Giguère, & Peretz, 2007; Pfordresher & Brown, 2007). Poor singing will be thoroughly examined in a separate section.

Occasional singers exhibit accurate memory of the initial pitch and tempo of popular songs (Bergeson & Trehub, 2002; Halpern, 1989; Levitin, 1994; Levitin & Cook, 1996) but poor vocal pitch-matching abilities (Amir, Amir, & Kishon-Rabin, 2003; Mürbe, Pabst, Hofmann, & Sundberg, 2002; Ternstrom, Sundberg, & Collden, 1988). When asked to reproduce single pitches in pitch-matching tasks, non-musicians deviate by 1.3 semit. (semitones) on average as compared to 0.5 semit. for musicians (Amir et al., 2003; Murry, 1990; Murry & Zwiner, 1991; Ternstrom et al., 1988). However, higher accuracy (i.e., with pitch deviations below 0.5 semit.) was found in non-musicians when the pitches to be imitated were synthesized voices or sung performances (Pfordresher & Brown, 2007; Wise & Sloboda, 2008). Moreover, pitch-matching is easier when the model is someone’s own voice as compared to a neutral female voice or non-vocal complex tones (Moore, Estis, Gordon-Hickey, & Watts, 2008; but see Price, 2000). These findings indicate that the measure of pitch accuracy in adults may depend on the characteristics of the model to be imitated, as previously observed in children (e.g., Green, 1990; Small & McCachern, 1983). Other studies focused on the relation between accuracy in pitch-matching tasks and pitch discrimination skills. For example, Watts and collaborators (Watts, Moore, & McCaghren, 2005) showed that pitch-matching in untrained singers co-varies with the ability to discriminate pitches (i.e., accurate singers are more accurate in discriminating pitches than less-accurate singers; see also Watts, Murphy, & Barnes-Burroughs, 2003). However, this relation between perception and performance is not confirmed by other studies (Bradshaw & McHenry, 2005; Moore et al., 2008). In summary, the extent to which pitch perception and production are related is still a subject of debate. We will return to this discussion in the section devoted to poor singing in tone deafness.

The imitation of intervals and short novel melodies by occasional singers was examined systematically in two recent studies. Pfordresher and Brown (2007) asked more than 100 university students to imitate short melodies of increasing complexity (i.e., a single repeated note, a sequence including a single change of pitch, and short four-note melodies). Most occasional singers were able to imitate sequences without transposing the pitch (i.e., within ± 1 semit. from the target pitches). They were less accurate, however, in reproducing the target pitch in the context of melodies (average deviation > 1 semit., Experiment 1), than with sequences including just one interval (deviation < 1 semit., Experiment 1). Their production of relative pitch was also affected by melody complexity, showing greater deviation from the target intervals with melodies (on average > 1 semit., Experiment 1) than with one-interval sequences (< 1 semit., Exp. 1). Moreover, occasional singers slightly compressed intervals (i.e., they produced smaller intervals than expected). Similarly, Wise and Sloboda (2008) asked 17 university students (self-defined not tone deaf) to imitate single pitch and patterns including two, three, or five pitches. Absolute deviation of the produced pitches from the targets increased with the number of elements in the sequence to be imitated. In summary, despite early suggestions that occasional singers are quite inaccurate in imitating single pitches, recent studies have yielded more optimistic results. Nonetheless, accuracy in imitating pitch rapidly decreases with increasing sequence length and complexity. 

A common behavior among occasional singers (e.g., more common than imitating single pitches or intervals) is to perform well-known songs from memory. Singing proficiency in producing familiar melodies is often assessed by peers (e.g., Alcock, Passingham, Watkins, & Vargha-Khadem, 2000; Alcock, Wade, Anslow, & Passingham, 2000; Hébert, Racette, Gagnon, & Peretz, 2003; Racette, Bard, & Peretz, 2006; Schön, Lorber, Spacal, & Semenza, 2004; Wise & Sloboda, 2008). However, discrepancies between subjective ratings are frequent (e.g., Kinsella, Prior, & Murray, 1988; Prior, Kinsella, & Giese, 1990). Indeed, perceptual constraints may impinge on peer judgments. Moreover, peers can hardly provide fine and independent estimates of accuracy in the dimensions of pitch and time. Acoustical methods represent a powerful alternative (e.g., Dalla Bella et al., 2007, 2009; Murayama, Kashiwagi, Kashiwagi, & Mimura, 2004; Terao et al., 2006). Features such as note pitch onsets and pitch height derived from the acoustical analysis of the recording afford objective and reliable measures of singing proficiency. In a study (Dalla Bella et al., 2007), occasional singers (20 university students tested in the lab and 42 participants recruited in a public park) were asked to sing a highly familiar song with lyrics. Acoustical analyses showed that pitch intervals were less accurately produced by occasional singers (with produced intervals deviating on average by 0.6 semit. from the melody notation) as compared to four professional singers (with interval deviation of 0.3 semit.). Occasional singers did not differ from professional singers in terms of temporal variability (herein referring to the produced note durations relative to the notation); still, on average, they sang faster than the professionals. Moreover, faster tempi were associated with lower pitch accuracy. To test the role of tempo in singing proficiency, 15 of the occasional singers were retested; they performed the same familiar melody as before, but at a slow tempo. Thirteen singers exhibited improved accuracy in the pitch dimension when they sang at a slower tempo. Their performance was comparable to that of the professional singers. However, 2 singers did not improve; thereby, they were qualified as *poor-pitch singers* (similar cases of poor singing will be discussed below). We recently replicated these results (Berkowska & Dalla Bella, 2009; Dalla Bella & Berkowska, 2009) in a group of 39 occasional singers tested using different familiar material. In addition, we found that imitating a familiar song at a slow tempo enhanced both pitch accuracy and reduced temporal variability. In summary, occasional singers are as accurate in producing pitch intervals and as temporally variable as professional singers, provided that the tempo is slow and that the melody to be imitated is presented together with a metronome.

A few studies have focused on the neuronal underpinnings of sung performance (mostly pitch production) in normal participants using neuroimaging techniques (i.e., PET and fMRI) and brain stimulation (i.e., TMS). Singing has often been contrasted with speech production (see Gordon, Racette, & Schön, 2006, for a review). Although there is a significant overlap of the areas recruited by singing and speaking, a predominant right-hemisphere involvement in vocal pitch performance, as opposed to left-hemisphere involvement in speech, is observed. For example, covert singing of well-known non-lyrical tunes has been associated with larger activation in the right sensorimotor cortex whereas speaking an over-learned word string engages the left sensorimotor cortex (Ackermann & Riecker, 2004; Riecker, Ackermann, Wildgruber, Dogil, & Grodd, 2000; Wildgruber, Ackermann, Klose, Kardatzki, & Grodd, 1996). A similar lateralization pattern involving for example the insula and the planum temporale was found when speaking and singing with lyrics were contrasted (Callan et al., 2006, with covert performance; Jeffries, Fritz, & Brown, 2003, with overt performance). In addition, when transcranial magnetic stimulation was applied over the left-hemisphere regions, traditionally related to speech production (e.g., near Broca’s area), speech was disrupted; similar stimulation over homologous brain areas in the right hemisphere affected singing (Epstein et al., 1999; Lo & Fook-Chong, 2004). Melody disruption subsequent to right frontal stimulation, however, did not occur in all participants (e.g., 2 out of 10 in Epstein et al., 1999). These findings point to more bilateral involvement in singing than in speech production (see also Brown, Martinez, & Parsons, 2006).

**Figure 1.**
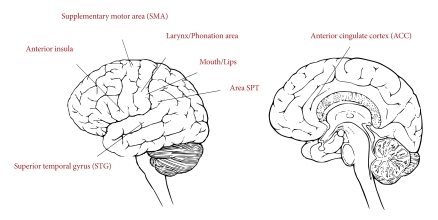
Neuronal underpinnings of the human song system.

Other neuroimaging studies have focused on the neuronal substrates of the human song system, uncovering a quite consistent functional network including motor and sensory areas as well as auditory-motor integration regions (see Figure 1). Singing recruits regions of the primary motor cortex, such as the mouth region (e.g., Brown, Martinez, Hodges, Fox, & Parsons, 2004), and the larynx/phonation area, activated by adduction/abduction and tension/relaxation of the vocal folds (Brown, Ngan, & Liotti, 2008). The larynx area, recently described, is likely to function as the major vocal center of the motor cortex in humans. The primary auditory cortex (i.e., the superior temporal gyrus, STG) is also engaged by vocal performance, for example when repeating a single note (Perry et al., 1999) or singing more complex melodies (Brown et al., 2004; Kleber et al., 2007). Other cortical areas which are systematically recruited by vocal performance are the supplementary motor area (SMA), the anterior cingulate cortex (ACC), and the insula (Brown et al., 2004; Kleber et al., 2007; Perry et al., 1999; Zarate & Zatorre, 2008). The SMA is notoriously engaged in high-level motor control, needed for efficient motor planning in sequence production, such as in overt speech production (e.g., Turkeltaub, Eden, Jones, & Zeffiro, 2002). The ACC is involved in the initiation of vocalization, as indicated by studies on primates (see Jürgens, 2002, for a review), and is implicated in overt speech and singing (Paus, 2001; Perry et al., 1999). Finally, the anterior insula is associated with vocalization processes, mostly articulation (e.g., Dronkers, 1996). Because the anterior insula is connected to both the ACC and to the auditory areas, this region may be involved in integrating auditory feedback with motor output (Ackermann & Riecker, 2004; Riecker et al., 2000).

Within this complex network, certain areas (e.g., the inferior sensorimotor cortex and the superior temporal gyrus and sulcus) are shared by speaking and singing (Gunji, Ishii, Chau, Kakigi, & Pantev, 2007; Özdemir, Norton, & Schlaug, 2006). These regions are likely responsible for auditory–motor integration, which is a key process in monitoring pitch in vocal performance (Zarate & Zatorre, 2008). More specifically, the area SPT (i.e., cortex of the dorsal Sylvian fissure at the parietal-temporal junction) is activated both during covert speech and covert humming (Hickok, Buchsbaum, Humphries, & Muftuler, 2003); this region is thought to function as a sensorimotor interface in speech production (Hickok, Okada, & Serences, 2009; Hickok & Poeppel, 2007). Audio-vocal integration in singing was recently examined in an interesting study by Zarate and Zatorre (2008) using altered auditory feedback. Non-musicians and experienced singers sang a single tone either when normal auditory feedback was provided or with pitch-shifted auditory feedback. Participants were instructed either to ignore the feedback or to compensate by pitch correction. Experienced singers, albeit more accurate in producing single pitches, recruited a very similar neural network to the one observed in non-musicians. In particular, this study suggests that the dorsal premotor cortex acts as a basic auditory–motor interface. Other cortical regions, such as the ACC and the auditory cortex, would be more involved as vocal training and practice increase. In summary, the reviewed studies point to a complex neuronal network underpinning vocal performance in singing, including sensory, motor, and sensorimotor integration areas. Yet, more research is needed to come up with a model indicating the connectivity of the areas underlying singing (for an example in speech, see Hickok & Poeppel, 2007), and their involvement in different singing tasks (e.g., single pitch-matching vs. singing a complex melody from memory). 

## POOR SINGING

The mechanisms underlying singing can be disrupted by brain damage (i.e., acquired disorders) or neurogenetic (i.e., congenital) disorders, thereby leading to poor singing. In two separate sections we will review the studies on poor singing consequent to brain damage and poor singing in the general population without brain damage (e.g., in tone deaf individuals). Before reviewing these studies, however, it is worth examining the criteria adopted to qualify individuals as poor singers. Indeed, even when singing accuracy is examined with objective acoustical methods, different criteria are used to define poor singing. In the majority of studies, poor singing mostly refers to inaccuracies on the pitch dimension, thus neglecting the time/rhythm dimension (e.g., Pfordresher & Brown, 2007; Welch, 1979; Wise & Sloboda, 2008; but see, e.g., Dalla Bella & Berkowska, 2009; Dalla Bella et al., 2007). Poor-pitch singers can be qualified as such based on a fixed criterion, for example when in a pitch-matching task their produced pitches depart from a target pitch by more than a semitone (e.g., Pfordresher & Brown, 2007). The alternative is to adopt a variable criterion. Individuals can be classified as poor-pitch singers relative to a control/comparison group, as often observed in single-case studies of patients with brain damage (e.g., Satoh, Takeda, & Kuzuhara, 2007; Schön et al., 2004). Another possibility is to consider poor-pitch singers as those individuals who are outliers in a given group (e.g., departing from the mean performance of the group by more than two standard deviations). This criterion has served in previous studies to determine whether individuals are congenital amusics, based on their performance in perceptual and memory tests (Peretz, Champod, & Hyde, 2003), and to define different phenotypes of poor singing in the general population (Dalla Bella & Berkowska, 2009). Finally, it is also worth noting that poor-pitch singing can be defined based either on absolute pitch measures (i.e., in pitch-matching and imitation tasks; e.g., Pfordresher & Brown, 2007), or on relative pitch measures (e.g., in singing from memory tasks, Dalla Bella et al., 2007). In summary, there is no single widely accepted set of criteria for defining poor singing. Thus, particularly when comparing results across studies, careful attention has to be paid to authors’ definitions of *poor singing*.

### Poor singing consequent to brain damage

Most studies of musical deficits consequent to a brain injury have addressed music perception. There are few systematic clinical reports of brain-damaged patients with expressive musical disorders, such as impaired singing (vocal amusia or oral-expressive amusia) or deficient musical performance on an instrument (expressive instrumental amusia or musical apraxia). These disorders have been generally referred to as *expressive amusia* (Benton, 1977). Cases of vocal amusias and instrumental amusias have been described since the XIXth century (Benton, 1977).

Impaired singing following brain damage has been reported in skilled professional singers and in non-musicians (for reviews, see Ackermann, Wildgruber, & Riecker, 2006; Gordon et al., 2006; Marin & Perry, 1999). Early case reports indicate that lesions of the right-hemisphere fronto-insular cortex disrupt the ability to sing, hum, or whistle a tune (Jossmann, 1926, 1927, and Mann, 1898, 1933, cited in Benton, 1977; Botez & Wertheim, 1959). For example, Mann (1898, cited in Benton, 1977) described the case of a professional singer, following injury of the right frontal lobe, with impaired ability to sing and whistle songs. In spite of dramatically impaired vocal expression, however, the patient could recognize familiar songs and did not show any signs of aphasia. Similar cases of musicians exhibiting poor singing without concomitant language disorders, and with relatively spared music perception and recognition were reported by Jossmann (1926, 1927, as cited in Benton, 1977) and Botez and Wertheim (1959) . These findings are consistent with the observation that unilateral inactivation of the right hemisphere (i.e., with the Wada test; see Gordon & Bogen, 1974) alters the ability to sing, hum, or whistle a tune, which is in line with the neuroimaging evidence mentioned above. Unfortunately, however, most of these case descriptions are anecdotal (i.e, they lack systematic assessment of musical production and perception skills). 

A list of more recent systematic group and case studies is reported in Table 1. The localization and extent of brain damage is very variable across the patients in the studies reviewed herein. Moreover, the tasks and the analysis methods adopted widely vary. Hence, drawing a clear map of the brain areas necessary for proficient singing based on these few studies is a challenging task. Nonetheless, some conclusions can be drawn regarding the involvement of the right and left hemispheres in singing. For example, hemisphere specialization for pitch and rhythm vocal production was examined by Alcock and collaborators (Alcock, Wade, et al., 2000) in patients with unilateral fronto-temporal left- or right-hemisphere lesions. Left-hemisphere patients exhibited impaired rhythm performance and perception and were less likely than right-hemisphere patients to sing a song with lyrics spontaneously. Yet, their ability to sing the correct pitch was spared. Right-hemisphere patients, in contrast, showed major difficulties in pitch production and perception tasks, with less impaired rhythm processing than left-hemisphere patients. Additional evidence confirming that the right hemisphere is necessary for pitch production comes from two recent single-case studies in which pitch accuracy was assessed with acoustical methods (Murayama et al., 2004; Terao et al., 2006).

**Table 1.** Reports of Impaired Singing in Brain-Damaged Patients

	Lesion	Perception	Singing			Singing analysis method
Reports			Overall performance	Pitch	Rhythm	
Kinsella et al. (1988)	15 patients (right CVAs)	nt	-	-	-	Peer ratings
	15 patients (left CVAs)	nt	-	-	-	
Prior et al. (1990), Experiment 2	15 patients (right CVAs)	nt	-	-	-	Peer ratings
	15 patients (left CVAs)	nt	-	-	-	
Confavreux et al. (1992), amateur singer	RH: anterior temporal gyrus, insula. Bilateral frontal operculum	+ pitch direction- rhythm discrimination- familiar melody recognition	-	nt	nt	Peer ratings
Alcock, Wade, et al. (2000)	13 patients with unilateral fronto-temporal LH lesions	+ pitch, - rhythm	- (songs with lyrics)	+	-	Peer ratings Acoustical method for single notes and oral rhythms
	14 patients with unilateral fronto-temporal RH lesions	- pitch, - rhythm	+ (songs with lyrics)	-	+	
Schön et al. (2004), singer	RH: inferior frontal gyrus, posterior temporal, inferior parietal	+	-	- pitch intervals	+	Peer ratings
Murayama et al. (2004), nonmusician	RH: frontal (superior, middle, inferior, and precentral gyri), superior temporal gyrus, insula, postcentral gyrus, inferior parietal lobule	nt	-	-	+	Acoustical method
Terao et al. (2006), singer	RH: superior temporal gyrus, supramarginal gyrus, posterior postcentral gyrus, posterior insula	- timbre, pitch, loudness	-	-	nt	Acoustical method
Satoh et al. (2007), nonmusician	LH: middle temporal gyrus RH: superior, middle, and inferior temporal gyri, transverse gyrus of Heschl, insula	- discrim./recognition familiar songs, unfamiliar phrases, chords	-	-	+	Ratings (?)

*Note*. CVAs = cerebrovascular accidents. + = normal. - = impaired. nt = not tested.

A classical interpretation of these findings is that singing familiar songs engages the right-hemisphere regions as opposed to the left-hemisphere involvement in processing propositional (generative) speech. This account is confirmed by the observation of the opposite dissociation between speech and music, showing preserved singing abilities in some patients with severe expressive aphasia (e.g., Amaducci, Grassi, & Boller, 2002; Assal, Buttet, & Javet, 1977; Hébert et al., 2003; Sparks, Helm, & Albert, 1974; Yamadori, Osumi, Masuhara, & Okubo, 1977). This evidence, however, is not clear-cut. Poor singing is often associated with linguistic deficits following left-hemisphere damage (e.g., Benton, 1977). Furthermore, evidence that lesions in either of the two hemispheres can affect singing accuracy (Kinsella, Prior, & Murray, 1988; Prior et al., 1990), that both right- and left-hemisphere anesthetization interfere with singing (Borchgrevink, 1980; Zatorre, 1984), that “singing seizures” in some epileptic patients are not clearly lateralized (e.g., Bentes, Pimentel, Costa, Santos, & Rolo, 2008; McChesney-Atkins, Davies, Montouris, Silver, & Menkes, 2003), and that singing without words does no elicit any lip-opening asymmetry, as a measure of laterality (e.g., Cadalbert, Landis, Regard, & Graves, 1994; Hough, Daniel, Snow, O’Brien, & Hume, 1994) rather suggests bilateral hemispheric involvement in singing.

Moreover, it can be observed that some recurrent lesional sites are also part of the human song system, as previously described. For example, lesions to the STG and to the insula are in most of the cases associated with impaired pitch production (e.g., Confavreux, Croisile, Garassus, Aimard, & Trillet, 1992; Murayama et al., 2004; Satoh et al., 2007; Terao et al., 2006). This observation confirms that these areas are relevant for proficient singing, as indicated by brain imaging data.

Finally, there is evidence that brain damage can selectively affect production while leaving perception relatively intact. For example, Confavreux and collaborators (1992) reported the case of a patient with focal cerebral degeneration (progressive amusia) of the right-hemisphere regions involving the anterior temporal gyrus and the insula. The patient, a poor singer, showed relatively spared pitch perception (i.e., with correct perception of pitch direction, but with deficient rhythm discrimination and melody recognition). Poor singing was accompanied by expressive aprosody. That impaired production can coexist with relatively spared perception consequent to brain damage was confirmed in a study by Schön and collaborators (2004). They reported the case of a tenor singer (IP) with right hemisphere lesions distributed in the inferior frontal gyrus, posterior temporal lobe, and inferior parietal lobe. IP is a pure case of expressive vocal amusia, exhibiting selectively deficient production of musical intervals. In contrast, IP’s production of rhythm and contour was spared, as well as his musical perception skills and language abilities.

To summarize, evidence from more systematic case and group studies indicates that, even though singing engages predominantly right-hemisphere structures, it is likely characterized by less strict lateralization than speech. This conclusion is in keeping with brain imaging studies. In addition, singing disorders can occur in a relatively pure form, in the absence of perceptual and linguistic deficits, and can concern very specific aspects of musical vocal production (e.g., interval production), while leaving other functions intact.

### Poor singing in tone deafness

Despite the fact that accurate singing is widespread in the general population, a few individuals have notorious difficulties in carrying a tune. These poor singers are thought to represent approximately 10-15% of the population (Dalla Bella & Berkowska, 2009; Dalla Bella et al., 2007). Poor singing is considered by the majority as a landmark of a more general lack of musicality, or tone deafness (see Sloboda et al., 2005, for a discussion). The widespread term *tone deafness*, albeit being ill-defined, literally suggests that poor singing may be the outcome of a deficient perceptual system. Indeed, lack of musicality has been mostly associated with poor perceptual abilities, a condition referred to more specifically as *congenital amusia* (Ayotte, Peretz, & Hyde, 2002; Foxton, Dean, Gee, Peretz, & Griffiths, 2004; Peretz, 2001; Peretz et al., 2002; Peretz & Hyde, 2003). Congenital amusia affects about 4% of the population (Kalmus & Fry, 1980; Peretz & Hyde, 2003), and has been shown to be hereditary (Peretz, Cummings, & Dubé, 2007). This condition is associated with brain anomalies in the right inferior frontal cortex (Hyde, Zatorre, Griffiths, Lerch, & Peretz, 2006), and in the right auditory cortex (Hyde et al., 2007). Individuals with congenital amusia exhibit mostly impoverished pitch perception (Ayotte et al., 2002; Foxton et al., 2004; Hyde & Peretz, 2004). This perceptual deficit is visible when amusics fail to discriminate pairs of melodies differing by a single note (Ayotte et al., 2002). Deficient pitch perception is likely to affect singing proficiency due to inaccurate auditory feedback.

The expected link between inaccurate pitch perception and poor-pitch singing was examined in a recent study (Dalla Bella et al., 2009). We tested singing proficiency in a group of 11 individuals with congenital amusia, as attested by the Montreal Battery of Evaluation of Amusia (MBEA; Peretz et al., 2003). The MBEA includes six tests. Three of them test the ability to discriminate changes in pairs of melodies, in terms of scale, contour, and interval size. Two tests serve to examine rhythm perception (i.e., rhythm discrimination and meter detection). The last task focuses on incidental musical memory. Amusics and matched control participants sang a highly familiar tune with lyrics from memory. Measures of pitch and time accuracy obtained with acoustical methods (as in Dalla Bella et al., 2007) showed that 9 out of 11 amusics were poor-pitch singers (e.g., they made several pitch interval errors and/or their performance lacked stability in terms of pitch). Five of them also sang out of time. It is particularly interesting that, when amusics were asked to sing the same familiar tune without lyrics (i.e., on one syllable), more than half of them could not sing more than a few notes. This contrasts with the performance of normal singers, who typically perform more in tune and more in time when singing without lyrics (Berkowska & Dalla Bella, 2009). This dissociation between singing with and without lyrics in amusics is likely to result from weak memory traces of the musical components of songs (e.g., Dalla Bella et al., 2009). The possibility that poor singing can result from memory deficits will be discussed in the next section. In addition, the amusics’ singing proficiency was correlated with their pitch discrimination abilities from a previous study (Hyde & Peretz, 2004): Amusics who were the least accurate in producing pitch intervals were also the most impaired in capturing pitch differences. Thus, these findings are in keeping with the hypothesis that there is a tight coupling between perception and action. However, note that the amusics’ pitch discrimination, albeit worse than in the controls, was still below one semitone; yet, the amusics were inaccurate at producing pitch intervals far above 1 semit. This suggests that poor low-level pitch discrimination cannot alone account for poor-pitch singing. Indeed, amusics are also deficient in tasks in which differences between intervals larger than one semitone are detected in a melodic context (e.g., Ayotte et al., 2002). This inaccurate pitch perception would hinder performance monitoring and error correction, thereby leading to poor singing; additionally, impoverished perception can account for the observation that congenital amusics are notoriously unaware of singing out of tune.

Deficient pitch perception, however, is not a sine qua non condition for poor singing. The simple observation that poor singing occurs more often (10-15%) in the general population than congenital amusia (4%) suggests that some individuals, despite normal perceptual abilities, may still be poor singers. This possibility is supported by a growing body of evidence that poor singing can co-occur with unimpaired perceptual abilities (Bradshaw & McHenry, 2005; Dalla Bella et al., 2007; Pfordresher & Brown, 2007; Wise & Sloboda, 2008). This condition has been referred to as *purely vocal tone deafness* (Dalla Bella et al., 2007). For example, in a group of 15 occasional singers we tested in the past, 13 participants sang proficiently at a slow tempo; in contrast, 2 participants were still very inaccurate in producing pitch intervals (Dalla Bella et al., 2007). These poor singers produced more than 10 inaccurate intervals (i.e., departing by more than 1 semit. from the intervals prescribed by the notation); moreover, their produced intervals deviated on average by at least 1 semit. from the notated intervals. The performance of these 2 poor singers sharply contrasts with singing in the remaining 13 participants, who made just a few interval errors (1.2 on average), and exhibited little deviation from the notated intervals (0.3 semit. on average). Yet, poor singing was not accompanied by impaired pitch perception: When asked to perform a task that required the detection of pitch and time incongruities in unfamiliar melodies (Peretz et al., 2008), these poor singers obtained 93% correct responses on average, a performance comparable to that of a group of university students (88% correct responses). Given unimpaired perception, thus, it is not surprising that these poor singers were fully aware that they did not sing in tune.

In another study, Pfordresher and Brown (2007) focused on poor pitch singing in the imitation of short novel melodies. Participants were defined as poor pitch singers when they transposed the pitches to be imitated by ± 1 semit. Of 79 participants, 10 (13%) were classified as poor pitch singers. Their poor accuracy in imitating pitch was not limited to pitch height (i.e., absolute pitch), but extended to the production of pitch intervals (i.e., relative pitch). Poor singers exhibited a marked tendency to compress intervals (i.e., they underestimated interval size during production), to a much greater extent than observed in proficient singers. Typically, poor singers both transposed and compressed intervals. In addition, they benefited from “choral singing” (i.e., when a synthesized voice was provided concurrently with the performance, indicating the correct pitch heights) in producing pitch intervals and melodic contour. Still, this additional feedback worsened their performance in terms of absolute pitch (i.e., more transposition was observed as compared to normal feedback). This finding contrasts with the performance of proficient singers, who capitalized on additional feedback to improve their accuracy in terms of both relative and absolute pitch. Interestingly, poor-pitch singers performed as accurately as proficient singers in a pitch discrimination task. Thus, as before, poor pitch singing could not be accounted for by impaired pitch discrimination abilities. Similar results were obtained by Wise and Sloboda (2008) , who tested the imitation of single pitch and short melodic patterns as well as perceptual and memory abilities with the MBEA in a group of 13 self-defined tone-deaf individuals. Tone-deaf individuals were less accurate in singing than a matched group of “not tone deaf” participants; this effect was more visible with longer stimuli. Unlike the findings of Pfordresher and Brown (2007) , however, errors in pitch imitation (i.e., the degree of transposition) were reduced when participants, including poor singers, sang along with the pattern to be imitated (i.e., choral singing). In spite of inaccurate pitch production, tone deaf individuals were comparable to not tone deaf participants using the MBEA. Again, this study reported cases of impaired singing which were not accompanied by perceptual deficits. 

The reverse dissociation (i.e., spared performance with deficient perception) is more paradoxical. Recent data, however, lend some support to this possibility. Loui and collaborators (Loui, Guenther, Mathys, & Schlaug, 2008) asked congenital amusics, identified based on their performance on the MBEA, to imitate tone intervals; in a second task, participants judged whether the second tone in a pair was higher or lower than the first. Like the controls, congenital amusics were able to reproduce pitch direction (ascending or descending). Nevertheless, they could not detect pitch direction, suggesting that there may be two separate streams for auditory perception and action (Griffiths, 2008). These results were partly replicated in a group of five congenital amusics who performed worse than the controls on a task of the MBEA requiring perception of pitch direction; still, they could produce the correct pitch direction when singing a melody from memory (Dalla Bella et al., 2009). Moreover, it is likely that this mismatch between perception and performance is not confined to pitch direction. Two amusics with severely deficient pitch perception were able to sing with lyrics as proficiently as the controls (Dalla Bella et al., 2009). In summary, there is a growing body of evidence pointing toward a double dissociation between perception and action mechanisms in congenital amusia/tone deafness (for a discussion, see Griffiths, 2008).

So far we have focused on dissociations between perception and action. Evidence has been provided that poor singing can be more or less associated with (or resulting from) perceptual disorders. New data is showing, however, that poor singing, instead of being a monolithic phenomenon, may not be a condition systematically involving all skills underlying proficient singing (i.e., there may be a diversity of poor singing “phenotypes”). For example, in a recent study we examined patterns of poor singing in a group of 39 occasional singers (Dalla Bella & Berkowska, 2009). The participants performed a battery of tests (Sung Performance Battery), ranging from single pitch-matching tasks to the imitation of well-known songs (e.g., *Brother John, Jingle Bells*) at a controlled slow tempo. Here we will focus only on the results obtained with the last task, which served to characterize different poor singing “phenotypes”. Acoustical measures afforded an estimate of accuracy on the pitch and time dimensions. For each dimension, accuracy was examined in absolute terms (i.e., amount of pitch transposition and tempo change), and in relative terms (i.e., accuracy in reproducing pitch intervals and relative durations). Participants were characterized as “poor singers” on a given dimension (e.g., the reproduction of interval size) if their performance lay beyond a cut-off score corresponding to the average value of that variable for the overall group plus two standard deviations. The found patterns were classified according to two axes: pitch vs. time accuracy and relative measures vs. absolute measures of accuracy (see Dalla Bella & Berkowska, 2009). The occasional singers were more inaccurate in terms of absolute measures than of relative measures. Of the tested population, 8% transposed pitch by more than 4 semit. (i.e., pitch transposers), without being inaccurate on the other dimensions. Another 8% (i.e., tempo transposers) sang faster or slower than the melody to be imitated (i.e., with performed tempo deviating by more than 10% from the target tempo), without transposing pitch. An additional 5% were inaccurate in producing interval size (i.e., poor pitch interval singers), deviating by more than 1 semit. on average from the notated intervals; in contrast, they displayed little transposition. Only 3% were selectively inaccurate in producing note relative durations (i.e., poor duration singers). Poor singers were more affected on the pitch dimension than on the time dimension, in keeping with previous findings (Dalla Bella et al., 2007, 2009). Dissociations along the pitch/time and absolute/relative measure axes indicate that components of the general ability to sing fractionate in poor singers. The mechanisms underlying pitch and time processing, and relative/absolute processing of pitch and time, may enjoy some degree of functional independence, a possibility which is discussed more thoroughly below.

## EXPLANATIONS OF POOR SINGING

**Figure 2.**
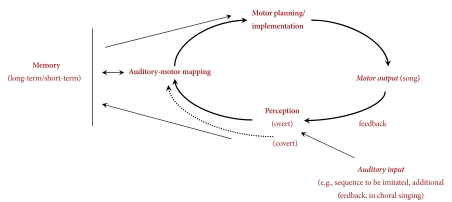
Vocal sensorimotor loop.

The dissociation between perception and action mechanisms in singing and the diversity of described phenotypes suggest that different sources of impairment can be responsible for poor singing. For example, deficient motor processing, inaccurate perception, malfunctioning sensorimotor integration mechanisms, or inaccurate memory can bring about poor singing (Pfordresher & Brown, 2007). To shed light on some of the mechanisms which are likely to be impaired in poor singers, we focus here on the components of the vocal sensorimotor loop, as schematically illustrated in Figure 2. This schema is inspired by previous models of performance monitoring and correction in speech, such as the Perceptual Loop Theory (Levelt, 1989). This theory specifies the monitoring systems active during speech performance, accounting for speakers’ attending to their own internal speech before uttering, as well as paying attention to their self-produced overt speech. Because similar processes characterize vocal performance in music, a description of the mechanisms underlying self-monitoring of performance appears to be a promising approach to account for accurate and inaccurate singing.

According to the presented schema, singing from memory of well-known melodies requires the retrieval of pitch and temporal information from long-term memory and fine motor planning/implementation. In addition, the ongoing vocal production is fed back to the system (i.e., perception), compared with the intended melody, thus eventually influencing motor planning (e.g., through error correction) for the subsequent note to be produced. Similar mechanisms are engaged in imitation. The target melody to be imitated is perceived, stored in the short-term memory, and the stored pitches mapped into motor gestures. As before, a feedback mechanism allows the singer to monitor his/her ongoing performance and to correct errors, if needed. Sometimes additional feedback can be provided, for example in the case of “choral singing”.

This simple schema is sufficient to account for some of the causes leading to poor singing. Poor singing can result from deficient perception, as observed in congenital amusia (e.g., Ayotte, et al., 2002; Dalla Bella et al., 2009; Foxton et al., 2004; Hyde & Peretz, 2004; Peretz et al., 2002; Peretz & Hyde, 2003). Impaired perception hinders appropriate monitoring of the ongoing overt performance, thereby leading to inadequate error correction and to diminished accuracy. In addition, due to this deficit in monitoring their own performance, congenital amusics are not aware of their deficit. However, observations of purely vocal tone deafness (e.g., Dalla Bella et al., 2007; Pfordresher & Brown, 2007; Wise & Sloboda, 2008) and of poor singing concurrent with spared perception following brain damage (Schön et al., 2004) suggest that in other cases the locus of impairment is past perceptual processes, rather involving sensorimotor integration (Pfordresher & Brown, 2007; see also Mandell, Schultze, & Schlaug, 2007) or memory retrieval/motor planning. The possibility that in some cases tone deafness is the outcome of malfunctioning or underdeveloped pathways bridging perception and action is supported by recent evidence of abnormally reduced connectivity of the fasciculus arcuatus (i.e., a pathway connecting temporal and frontal brain areas) in tone-deaf individuals (Loui, Alsop, & Schlaug, 2009).

Yet, impoverished perception does not mandatorily affect singing accuracy. Congenital amusics can exhibit spared production (Dalla Bella et al., 2009; Loui et al., 2008) despite dramatically impaired perception. This intriguing finding has been taken as evidence in favor of two separate streams for auditory perception and action (Griffiths, 2008), thus extending to the auditory modality the idea of independent perceptual and action systems previously observed in vision (i.e., dorsal and ventral systems, Goodale et al., 1991). This dissociation is reminiscent of action-blindsight in vision (e.g., Danckert & Rossetti, 2005, for a review) where the lack of awareness of visual stimuli does not preclude implicit treatment of information by the visual system (e.g., sufficient for spatial localization by pointing or saccading toward the stimuli). A possible reason for spared production in some cases of congenital amusia is that accurate performance of certain musical features (e.g., pitch direction) may not require overt perception (e.g., as measured in pitch discrimination tasks); covert perception (indicated by the dotted line in Figure 2), recruiting a separate pathway than the one engaged by overt perception, would be sufficient. The possibility of covert perceptual feedback mechanisms in vocal performance has received support from a recent study with altered auditory feedback in trained singers. When altered feedback (i.e., pitch-shifted voice provided 2 s after the participants produced a single note) was not perceptible, singers still reacted by changing the produced pitch height in the opposite direction (Hafke, 2008).

Another potential cause of poor singing pertains to memory. Weak memory traces, underspecified representation of song structure in long-term and/or short-term memory, or impaired access to long-term information can hinder proficient singing (e.g., Pfordresher & Brown, 2007; Wise & Sloboda, 2008). In the vocal sensorimotor loop, memory processes are generally supposed to function in parallel (and to interact) with auditory–motor mapping, while receiving input from perceptual processes and affecting/directing motor planning. Memory as a reason for poor singing has recently received some support. The finding that congenital amusics with particularly poor incidental memory for music are unable to sing a well-known melody on a syllable (Dalla Bella et al., 2009) is compatible with the memory explanation. Retrieving melody information from the long-term memory and associating it with new speech segments (e.g., a repeated syllable) is likely to be too challenging for amusics, who may prefer to rely on a compound music/lyrics code. Another piece of evidence in favor of a memory explanation is that singing along with the pattern to be imitated alleviated pitch production deficits in tone-deaf individuals (Wise & Sloboda, 2008; but see Pfordresher and Brown, 2007, who failed to replicate this effect). In summary, although memory factors appear to play a role in poor singing, it is still unclear to what extent this is the case, and whether poor singing can be accounted for by isolated memory disorders (i.e., in absence of perceptual, motor, and sensorimotor deficits).

Further patterns of poor singing (Dalla Bella & Berkowska, 2009) suggest additional subdivisions within the vocal sensorimotor loop. That singing can be selectively inaccurate in terms of pitch or time raises the possibility that these two dimensions may be processed separately in production. Note that independence of pitch and rhythm mechanisms in perception is supported by the study of patients with brain damage (e.g., Peretz, 2001; Peretz & Coltheart, 2003). In performance, there is a paucity of studies contrasting pitch accuracy to time accuracy. Hence, further enquiry is needed to clarify whether pitch and time production engage different mechanisms, and to determine the locus within the vocal sensorimotor loop where these two dimensions are treated separately, beyond perception. In addition, the dependence of singing proficiency on tempo (Dalla Bella & Berkowska, 2009; Dalla Bella et al., 2007) will need to be accounted for within the framework of the vocal sensorimotor loop.

In particular, pitch transposition and interval compression have been associated with “sensorimotor mistranslation” during imitation (Pfordresher & Brown, 2007), referring to inaccurate mapping of auditory representation to motor representations for phonation. This phenomenon may concern the reproduction of local musical features (absolute pitch and secondarily pitch intervals) without affecting global features like melodic contour (see Pfordresher & Brown, 2007). Yet, the dissociations recently observed in poor singers between absolute and relative measures of pitch/time accuracy (Dalla Bella & Berkowska, 2009) suggest that the mechanisms underpinning the production of absolute and relative musical features may enjoy a certain degree of independence. A consistent transposition error (e.g., in the pitch domain) may result from faulty linear auditory-motor mapping. Yet, this can hardly account for more complex patterns of pitch interval errors (e.g., departures from simple compression) or time errors as they involve more complex mapping rules (e.g., non-linear) and probably engage other mechanisms during memory retrieval and motor planning. The possibility that the production of absolute and relative musical features may engage at least partly independent mechanisms is supported by differential effects of feedback on pitch accuracy (i.e., choral singing enhances pitch accuracy in producing intervals and contour, but is detrimental to producing absolute pitch; Pfordresher & Brown, 2007). Further research is required to clarify which mechanisms within the vocal sensorimotor loop are responsible for processing absolute and relative information.

## CONCLUSIONS

In the present article we reviewed studies focusing on singing abilities in the general population. Increasing evidence indicates that occasional singers can sing proficiently, thus contradicting the widespread belief that the majority of people cannot carry a tune. A minority exhibit poor singing, following brain damage or resulting from neurogenetic disorders (e.g., congenital amusia). Despite the paucity of research devoted to poor singing in adult occasional singers, the evidence to date is sufficient to draw several hypotheses to be examined in future studies. The study of inaccurate singing reveals interesting patterns of impairment which can shed light on the functioning of the human song system. For example, deficits of sung performance can be very specific, selectively affecting particular musical abilities (e.g., absolute pitch imitation or the production of pitch intervals). Each of these deficits defines a given poor singing phenotype and reflects the malfunctioning of some dedicated mechanisms within the human song system. The reported findings point to a complex system (herein referred to as vocal sensorimotor loop) underlying proficient singing, involving perceptual and motor planning components, memory retrieval, auditory–motor mapping, and complex feedback mechanisms. There is a need for further research in this area, which will contribute to elucidating the structure of the vocal sensorimotor loop and the role of each component in proficient singing and in poor singing. This will ultimately provide useful information for understanding the beneficial effect of vocal performance in rehabilitation (e.g., Götell, Brown, & Ekman, 2003; Racette et al., 2006; Schlaug, Marchina, & Norton, 2008; Tamplin, 2008).
